# Effect of walking with an active ankle exoskeleton on the biomechanical responses of the lumbar spine

**DOI:** 10.3389/fbioe.2025.1654585

**Published:** 2025-09-25

**Authors:** Jose E. Rubio, Junfei Tong, Aravind Sundaramurthy, Anup Pant, Sridevi Nagaraja, Meredith K. Owen, Michael A. Samaan, Brian Noehren, Jaques Reifman

**Affiliations:** ^1^ Department of Defense Biotechnology High Performance Computing Software Applications Institute, Defense Health Agency Research & Development, Medical Research and Development Command, Fort Detrick, MD, United States; ^2^ The Henry M. Jackson Foundation for the Advancement of Military Medicine, Inc., Bethesda, MD, United States; ^3^ Department of Physical Therapy, University of Kentucky, Lexington, KY, United States; ^4^ Department of Kinesiology and Health Promotion, University of Kentucky, Lexington, KY, United States

**Keywords:** low-back injury, exoskeleton, load carriage, musculoskeletal model, lumbar biomechanics

## Abstract

**Objective:**

Musculoskeletal injuries pose a health threat to U.S. Service members. In particular, the physical demands of walking and running with load carriage contribute to a high incidence of musculoskeletal injuries of the lower back. Active ankle exoskeleton devices are promising technologies that may help mitigate the impact of load carriage on the incidence of these injuries. However, the safe extended use of these devices requires an understanding of their beneficial or detrimental effects on the lumbar spine. In this pilot study, we investigated the impact of walking with an ankle exoskeleton device on lumbar biomechanical responses.

**Methods:**

We collected motion-capture data and computed tomography images for five young, healthy men walking with a 22.7 kg (50-lb) load for 5 km at a speed of 1.3 m/s, with and without an active ankle exoskeleton (ExoBoot EB60). We developed individualized musculoskeletal and finite-element models to characterize the effects of walking distance and ExoBoot use on the trunk flexion angle, joint reaction force at the L4-L5 joint, and stress on the L4-L5 intervertebral disc annulus.

**Results:**

While not statistically significant, we found that the peak trunk flexion angle and the peak annulus stress increased by 16% and 12%, respectively, after walking 5 km with the ExoBoot, and by 34% and 25%, respectively, without it. Similarly, the peak L4-L5 joint reaction force minimally increased by 4% with the ExoBoot, while it increased by 22% without the device.

**Conclusion:**

ExoBoot use likely attenuates the effect of fatigue on the lumbar spine induced by walking with load carriage.

## 1 Introduction

Musculoskeletal injuries are the leading cause of medical encounters, discharges, and chronic disabilities in the U.S. military, with the lower back being one of the most frequently injured sites (Gun et al., 2021; Pav et al., 2024). In fact, a retrospective study of active-duty Service members in 2021 reported that musculoskeletal injuries ranked first among medical conditions leading to hospitalization or ambulatory visits, including over 1.1 million medical encounters associated with back injuries, the majority of which were diagnosed as low-back (i.e., lumbar) pain (Armed Forces Health Surveillance Division, 2022). Similarly, a surveillance study reported that between 2001 and 2011 the incidence rate of intervertebral disc degeneration among active-duty Service members more than doubled, with concomitant increases in medical encounters and lost duty days (Armed Forces Health Surveillance Division, 2012). Notably, the incidence of these low-back injuries may be exacerbated by walking and running with a load, which induce changes in gait biomechanics and have higher energy demands compared to activities without load carriage (Knapik et al., 2004; Brown et al., 2014; Orr et al., 2014; Boffey et al., 2019). Nevertheless, Service members often carry loads that exceed 20 kg (∼44 lb) and may march up to 56 km (∼35 miles) per day during training (Department of the Army, 2022). Therefore, the high physical demands of walking and running with a load during military operations and the associated incidence of low-back injuries require solutions that could help mitigate the risk of musculoskeletal injuries.

Lower-body exoskeletons have the potential to reduce the impact of load carriage on the risk of musculoskeletal injury by augmenting the ability to walk and run with a load while lowering energy demands. In particular, active ankle exoskeletons, which use powered actuators to provide torque at the ankle joint, have been shown to reduce the energy requirements of walking by as much as 39%, walking with a load by 15%, and running by 27% compared to performing the same activities unassisted (Malcolm et al., 2013; Mooney et al., 2014a; Mooney et al., 2014b; Zhang et al., 2017; Sawicki et al., 2020). However, the use of these exoskeleton devices may also induce changes in gait biomechanics, including alterations in joint kinematics (angles and range of motion) and kinetics (forces and moments), which have not been characterized. For example, prior studies have shown that a reduction in stride length and hip extension moment decrease the mechanical demand in the lower legs when walking unassisted and without load carriage (Leteneur et al., 2013; Antos et al., 2022). However, it is not known whether exoskeleton devices can induce similar changes in gait biomechanics.

The current understanding of how ankle exoskeleton devices affect gait biomechanics is limited because almost all of the studies to date have focused on how these devices impact the human body over short time periods (e.g., less than 20 min, with a maximum walking distance of ∼1.8 km) (Mooney et al., 2014a; Mooney et al., 2014b; Mooney and Herr, 2016), excluded load carriage (Malcolm et al., 2013; Mooney et al., 2014a; Mooney and Herr, 2016), or did not measure or compute kinetics or kinematics parameters (Ingraham et al., 2022; Medrano et al., 2022; Peng et al., 2022; Hybart et al., 2023; Wu et al., 2024). The only exception is the recent study by Hybart and Ferris, who investigated changes in joint angle trajectories during exoskeleton-assisted walking at the ankle, knee, and hip for 30 min (walking distance of ∼2.1 km) and found no significant differences in kinematic parameters compared to unassisted walking (Hybart and Ferris, 2023). Overall, these studies overlooked the fact that acute muscle fatigue alters gait biomechanics and negatively affects risk factors of lower-body injury, such as a decrease in hamstring-muscle strength, which makes the human body more susceptible to injury when fatigued (Verschueren et al., 2020). In addition, these studies only investigated the impact of exoskeleton use on the gait biomechanics of the lower body, neglecting its effects on the lower back. As a result, it is unclear how walking distance and load carriage exceeding 30 min influence exoskeleton-induced changes in both lower-body and low-back biomechanics.

To address this limitation, we previously investigated the impact of an active ankle exoskeleton (ExoBoot EB60; Dephy Inc., Boxborough, MA) on lower-body gait biomechanics (Nagaraja et al., 2025), where we found that the use of ExoBoot induced beneficial alterations in stride length as well as in hip flexion and extension moments, after walking for 5 km. In this pilot study, we expanded our previous work by quantifying its effects on the low-back biomechanical responses of young, healthy men after walking with load carriage for 5 km. To this end, we developed a computational framework consisting of individualized musculoskeletal and finite-element (FE) models. Then, using these models, we predicted the temporal changes in joint kinematics, joint kinetics, and muscle forces in the entire body as well as the stress distribution in the L4-L5 intervertebral disc for each participant (N = 5) after a 5-km walk (∼60 min) while carrying a 22.7 kg (50-lb) load, with and without the ExoBoot. We hypothesized that the peak trunk flexion angle, the peak reaction force at the L4-L5 joint, and the peak stress in the L4-L5 intervertebral disc would each increase with walking distance irrespective of ExoBoot use. In addition, based on our previous work (Nagaraja et al., 2025), we hypothesized that walking with the ExoBoot would benefit the user by reducing the magnitude of these increases. As such, this pilot study broadened the scope of previous assessments of ankle exoskeleton devices by quantifying the impact of ExoBoot use on an unexamined body site during walking periods of ∼60 min. The knowledge gained in this study shall provide initial guidelines for the safe and effective use of the ExoBoot in extended military operations.

## 2 Methods

### 2.1 Participants and data collection

We enrolled young, healthy men (18–25 years old) with anthropometric measurements representative of U.S. Army recruits (Gordon et al., 2014). We included participants who self-reported being experienced treadmill walkers and free of musculoskeletal injuries for at least 3 months before participating in the study. Each participant signed an informed consent prior to data collection with the study protocol approved by both the University of Kentucky Institutional Review Board (Lexington, KY) and by the Office of Human Research Oversight of the U.S. Army Medical Research and Development Command, Fort Detrick, MD.

**FIGURE 1:**
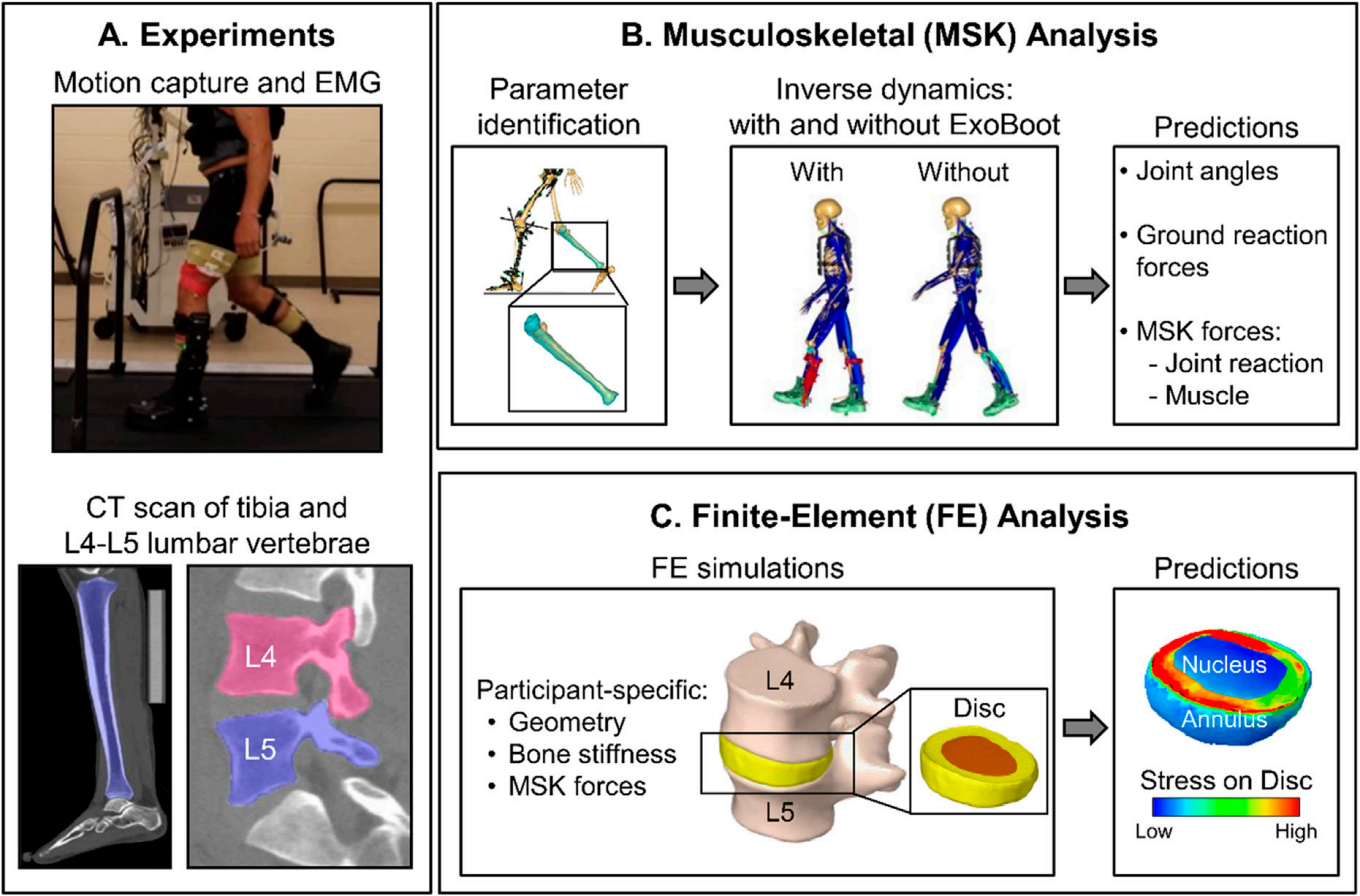
Summary of the integrated experimental and computational study design. **(A)** We performed a laboratory study and collected motion-capture (using 52 reflective markers), ground reaction force (GRF), and electromyography (EMG) data from five participants as they walked 5 km on a level treadmill with and without an active ankle exoskeleton device (the ExoBoot), with and without a 22.7 kg (50-lb) load. We collected EMG, GRF, and motion-capture data for 20 s at baseline (i.e., 0 km) and at each 1-km mark thereafter. We also collected computed tomography (CT) scans of the tibia and the L4-L5 lumbar vertebrae from each participant. **(B)** We developed individualized musculoskeletal models by incorporating participant-specific anthropometric, tibial, and lumbar spine data, and then used the models to perform simulations for two conditions, walking with and without the ExoBoot while carrying a 22.7 kg (50-lb) load. We used the simulation results to compute the GRFs, joint reaction forces (JRFs), joint angles, and joint moments for each participant under each walking condition. **(C)** For each participant, we developed finite-element (FE) models of the L4-L5 lumbar segment using participant-specific geometry and material properties for the vertebrae obtained from the CT scans. Next, we performed FE simulations using the joint and muscle forces from the musculoskeletal analyses as participant-specific loading conditions (i.e., inputs) to the model and predicted the stress distribution throughout the corresponding intervertebral disc (nucleus and annulus).

For each participant, we recorded their anthropometric measurements and collected computed tomography (CT) axial-plane images of the left tibia and L4-L5 lumbar vertebrae (Table 1; Figure 1A). We collected CT scans only for these two body sites because they are associated with an elevated risk of stress fracture, intervertebral disc degeneration, and low-back pain (Kardouni et al., 2021; van der Graaf et al., 2023; Bassani et al., 2024). We obtained the scans using a Siemens CT scanner (Siemens Medical Solutions, Malvern, PA) with an in-plane pixel resolution of 0.49 × 0.49 mm^2^ and a slice thickness of 0.60 mm. Each CT scan included a calibration phantom with six inserts of known calcium hydroxyapatite (HA) concentration (QRM, Moehrendorf, Germany) in the field of view, which allowed us to convert CT Hounsfield unit (HU) measurements into bone apparent density, as follows. First, for each CT scan, we measured the mean HU of each insert. Then, we plotted the known HA concentration of each insert as a function of its measured mean HU. Finally, we fitted a linear regression model to estimate the bone apparent density (in HA concentration units) from any measured HU in the CT scan.

**TABLE 1:** Anthropometric characteristics of 5 young, healthy men.

	Age (years)	Mass (kg)	Height (m)	Body fat (%)	BMI (kg/m^2^)
Mean (±SD)	22.0 (1.2)	85.2 (9.5)	1.77 (0.04)	15.3 (6.5)	27.1 (2.5)
Range	21–24	77.8–101.3	1.71–1.81	6.1–23.0	25.2–31.3

The data are presented as mean [±1 standard deviation (SD)] or a range. BMI: body mass index.

Each participant completed four walking trials in the same order over four separate visits. Each trial involved walking at a constant speed of 1.3 m/s for 5 km (∼60 min) on a flat-level, dual-belt instrumented treadmill (Bertec Corporation, Columbus, OH). Participants completed the trials in the following sequence: 1) walking without any additional load and without wearing an exoskeleton, 2) walking with a 22.7 kg (50-lb) load (V-max vest, Rexburg, ID) and without wearing an exoskeleton, 3) walking without any additional load while wearing an active exoskeleton (the ExoBoot EB60), and 4) walking with a 22.7 kg load while wearing the ExoBoot. In addition, prior to the first trial with the ExoBoot (i.e., visit 3), each participant completed a training visit to familiarize themselves with walking while wearing the ExoBoot. During this visit, we adjusted and aligned the ExoBoot shin pad to rest comfortably just below the kneecap of each participant. Moreover, to ensure that the participants were acclimated to walking with the ExoBoot on a force-plate instrumented treadmill, we allowed them to walk until they reported being comfortable with the device’s assistance for a minimum of 10 min. For the trials with load carriage, participants wore a V-max vest made of nylon with moisture-resistant padded foam and loaded with 50 lb of iron weights divided into 2.5-lb bars. They strapped the vest to their upper body, with the load symmetrically distributed between the front and back, in accordance with recommendations for military foot marches (Department of the Army, 2022). In addition, we selected the walking speed and load carriage to represent foot marches conducted by the U.S. Army (Department of the Army, 2022) and chose the ExoBoot because it is an ankle exoskeleton that has been shown to reduce metabolic cost during walking (Mooney et al., 2014b; Ingraham et al., 2022).

During each walking trial, we synchronously measured ground reaction forces (GRFs), motion-capture data, and electromyography (EMG) data for ∼20 s at baseline (i.e., 0 km) and at each 1-km mark thereafter (Figure 1). We measured GRFs from the instrumented treadmill at a sampling frequency of 2,000 Hz and collected EMG data with the same frequency from eight major lower-extremity muscles (gluteus medius, biceps femoris, vastus lateralis, rectus femoris, vastus medialis, gastrocnemius, soleus, and tibialis anterior), using preamplifier electrodes (Trigno Avanti, Delsys Inc., Natick, MA) placed bilaterally on locations recommended by the Surface EMG for Non-Invasive Assessment of Muscles (SENIAM) program (Hermens et al., 2000). In addition, we placed 52 reflective markers at several anatomical landmarks (head, torso, back, arms, and legs) and collected motion-capture data at a sampling frequency of 200 Hz using a 13-camera system (Motion Analysis Corporation, Santa Rosa, CA). As described in our previous work (Nagaraja et al., 2025), in the trials where participants wore the exoskeleton device, we placed some of the markers on the ExoBoot to track the motion of the lower leg. Finally, we continuously collected velocity, acceleration, and torque data measured by the ExoBoot throughout the entire duration of each walking trial at a sampling frequency of 200 Hz.

### 2.2 Computational analyses

#### 2.2.1 Individualized musculoskeletal models

As previously described (Xu et al., 2016; Xu et al., 2020; Unnikrishnan et al., 2021; Rubio et al., 2023; Sundaramurthy et al., 2023; Tong et al., 2023; Nagaraja et al., 2025), we developed and validated individualized musculoskeletal models using the AnyBody Modeling System (AnyBody Technology, Aalborg, Denmark) to predict temporal changes in joint kinematics (i.e., angles), joint kinetics (i.e., forces and moments), and muscle forces for each participant (Figure 1B). Briefly, we used the AnyBody-provided generic model of an average male consisting of rigid segments, including arms, trunk, pelvis, thighs, shanks, and feet, as well as 169 muscles in the lower extremities and 188 muscle fascicles in the trunk. To individualize the generic model to each participant, we first used the Mimics software (Materialise, Leuven, Belgium) to extract subject-specific geometries of the tibia and the L4-L5 lumbar vertebrae from the corresponding CT scan. Then, we morphed the generic tibial and L4-L5 lumbar vertebral geometries in the AnyBody model to match participant-specific geometries. Next, we scaled the length and mass of the remaining body segments in the generic musculoskeletal model using the anthropometric measurements (e.g., body mass, height, and body-fat percentage) of each participant. We further optimized the length of the body segments and joint centers using an optimization scheme that minimized the error between the experimentally tracked and modeled marker positions during a static pose (Andersen, 2021). In addition, as described in our previous work (Nagaraja et al., 2025), for trials that involved participants carrying a load or wearing the ExoBoot, we modeled the vest and the exoskeleton as rigid body segments in AnyBody. Briefly, we modeled the same vest as in the laboratory experiment, with straps connecting the vest to the body of the musculoskeletal model via two points at the clavicle. Similarly, we modeled the ExoBoot as three rigid body segments with a total weight of 12 N, using mass and inertia properties provided by the manufacturer.

We used the 20-second motion-tracking data collected for each walking trial and the participant-specific musculoskeletal models to compute the temporal changes in joint kinematics, joint kinetics, and muscle forces. In terms of kinematics, we computed the trunk flexion angle and the joint angles at the hip, knee, and ankle by implementing an optimization scheme to minimize the error (i.e., the distance) between the markers tracked during the experiment and the markers defined in the musculoskeletal model. In terms of kinetics, we computed the joint reaction forces at the L4-L5 lumbar vertebrae, hip, knee, and ankle as well as the external moments at the lower-extremity joints by conducting inverse dynamics analyses (Andersen, 2021). We normalized the GRF and the joint reaction forces by the participant’s body weight and normalized the joint moments by their body mass, because these normalization procedures have been shown to significantly reduce the variability introduced by height and weight (Moisio et al., 2003; Wannop et al., 2012). Finally, we conducted stride analyses to evaluate the output of the musculoskeletal simulations, as follows. We first identified the frames corresponding to separate strides by setting a threshold value of 25 N for the GRF at the start of a stride, resulting in 18–20 strides for each 20 s of data collected at each 1-km mark. Then, we resampled the biomechanical responses (i.e., both kinetics and kinematics) corresponding to a particular stride in order to define 100 equidistant values for each stride (Rubio et al., 2023; Nagaraja et al., 2025).

#### 2.2.2 Individualized finite-element models of the L4-L5 lumbar segment

We developed participant-specific FE models of the L4-L5 lumbar segment to predict the stress distribution in the intervertebral disc. We focused our study on this lumbar segment because it has been associated with a high incidence of intervertebral disc degeneration and low-back pain, which are medical conditions relevant to military personnel who often walk and run with load carriage (Rodriguez-Soto et al., 2017; van der Graaf et al., 2023; Bassani et al., 2024; Pav et al., 2024). First, based on the L4-L5 lumbar vertebral geometry extracted from the CT scans, we reconstructed the geometry of the intervertebral disc by manually tracing its boundaries and using three-dimensional (3-D) interpolation schemes available in the Mimics software. Then, using the L4-L5 lumbar vertebral and disc geometry, we developed the corresponding 3-D FE meshes consisting of 10-noded quadratic tetrahedral elements using the HyperMesh software (Altair Engineering, Inc., Troy, MI).

We defined participant-specific material properties for the L4-L5 lumbar vertebrae FE model by using the Hounsfield units of the CT scans to determine the Young’s modulus (E) of each mesh element and assuming that each element was linear elastic and isotropic (Table 2). Based on the Young’s modulus, we classified each element as either bone marrow tissue (E < 6 MPa) or bone (E ≥ 6 MPa) (Rho et al., 1993). We assigned a Poisson’s ratio of 0.167 to the bone-marrow tissue elements and 0.325 to the bone elements (Sandino et al., 2015). Next, we defined the intervertebral disc nucleus as an incompressible, linear elastic and isotropic material (Xu et al., 2022) and represented the intervertebral disc annulus as an incompressible, anisotropic hyperelastic material, using the Holzapfel-Gasser-Ogden model. We obtained the material properties of the disc annulus from biaxial tensile tests on cadaver specimens (O'Connell et al., 2009; O'Connell et al., 2012; Momeni Shahraki et al., 2015; Xu et al., 2022).

**TABLE 2:** Material properties of the different components of the finite-element model of the L4-L5 lumbar segment.

Component	Density (kg/m^3^)	Material model	Elastic constants		Anisotropic hyperelastic constants					Source
			Young’s modulus (MPa)	Poisson’s ratio	C_10_ (MPa)	D (1/MPa)	K_1_ (MPa)	K_2_	Fiber dispersion parameter, κ	
Vertebra bone	1,660	Linear elastic	Participant-specific	0.325	–	–	–	–	–	CT scan
Vertebra bone marrow	1,006	Linear elastic	Participant-specific	0.167	–	–	–	–	–	CT scan
Intervertebral disc nucleus	1,130	Linear elastic	1.0	0.495	–	–	–	–	–	Xu et al. (2022)
Intervertebral disc annulus	1,130	Anisotropic hyperelastic	–	–	0.85	0.59	2.80	90	0.15	O'Connell et al. (2009), O'Connell et al. (2012), Momeni Shahraki et al. (2015), Xu et al. (2022)

CT: computed tomography.

We performed FE simulations to predict the stress distribution in the L4-L5 intervertebral disc for each participant and walking trial (Figure 1C). To this end, we applied the muscle forces and joint forces and moments computed from the musculoskeletal analyses as participant-specific loading conditions (i.e., inputs) to the L4-L5 lumbar segment model, where we coupled the muscle insertion points to the outer surface of the lumbar FE mesh. To optimize the computational wall-clock time, instead of using the entire 20-second (∼18–20 strides) output from the musculoskeletal analyses as input to the FE model, we computed the ±95% confidence intervals of each muscle force, joint force, and moment over the ∼18–20 strides, and used the confidence interval values as inputs, resulting in only two simulations per participant for each walking condition. Using the ABAQUS 2022 software (Dassault Systèmes, Vélizy-Villacoublay, France), we performed FE analyses and computed the von Mises stress (95th percentile) on each element of the L4-L5 intervertebral disc, which is a relevant biomechanical response that accounts for the multiaxial stress state in the intervertebral disc (Motiwale et al., 2018; Subramani et al., 2020).

#### 2.2.3 Validation of computational models

To evaluate the validity of the individualized musculoskeletal models, we qualitatively compared the time courses of the predicted muscle activity with the EMG recordings (Nagaraja et al., 2025). In addition, we assessed the ability of a participant-specific FE model to predict the biomechanical responses of the L4-L5 intervertebral disc to pure compression by comparing model predictions with experimental data available in the literature (Markolf and Morris, 1974; Adams et al., 1994; Adams et al., 1996). We investigated the response to compressive loads because walking with a load significantly increases the peak compressive load on the lumbar spine (Goh et al., 1998; Li et al., 2019), which in turn acts primarily on the intervertebral discs. To mimic the experimental conditions, we defined two boundary conditions in the FE simulations by: 1) setting the displacement to zero at the bottom facet of the L4 vertebra and 2) defining a uniform compression force at the top facet of the L5 vertebra.

### 2.3 Statistical analysis

As previously described (Nagaraja et al., 2025), we conducted a power analysis to estimate the sample size required to detect changes in biomechanical responses during walking with and without the ExoBoot. Based on Gregorczyk et al. (2010), we estimated an effect size (ES) of 0.75 for kinematic and kinetic responses resulting from exoskeleton-assisted marching for 8 min. Therefore, assuming a larger ES of 1.00 because of the longer duration of our study (8 vs 60 min), a correlation among repeated measurements of 0.5, and a non-sphericity correction of 1.0, we determined that we needed a sample size of five participants to achieve 80% power at a 5% significance level.

To assess the impact of walking with or without the ExoBoot and walking distance on the biomechanical responses of the L4-L5 lumbar segment, we developed linear mixed-effects models for each biomechanical response (i.e., trunk flexion angle, L4-L5 joint reaction force, and L4-L5 intervertebral disc annulus stress), where we included three fixed categorical effects (i.e., device, distance, and device-distance interaction) and a random intercept to account for within-participant variability. First, we evaluated the significance of the interaction term in the model by performing a likelihood ratio (LR) test between a model with the interaction and one without it. When the interaction term was not statistically significant, we assessed the impact of each categorical effect on the biomechanical responses by performing univariate analyses using LR tests between a null model (i.e., no fixed effects) and a linear mixed-effects model with a single categorical effect and a random intercept. Finally, we computed the ES and performed pairwise comparisons between averages over the five participants for the two effects, using the Wilcoxon non-parametric signed-rank test with Holm-Bonferroni correction. We presented all data as mean ± one standard deviation (SD), unless otherwise noted. We used the RStudio v1.4 statistical software (R Core Team, 2023) for all statistical analyses with an alpha level of 0.05.

## 3 Results

### 3.1 Validation of computational models

We previously validated the participant-specific musculoskeletal models by qualitatively comparing the model-predicted muscle activities with the experimentally measured EMG recordings at 0 km for walking without the ExoBoot and no load (Nagaraja et al., 2025). With some exceptions, the musculoskeletal models adequately predicted the time frame when a particular muscle group (i.e., tibialis anterior, rectus femoris, or gluteus medius) was most active during a walking stride (Nagaraja et al., 2025) (Supplementary Figure S1).

**FIGURE 2:**
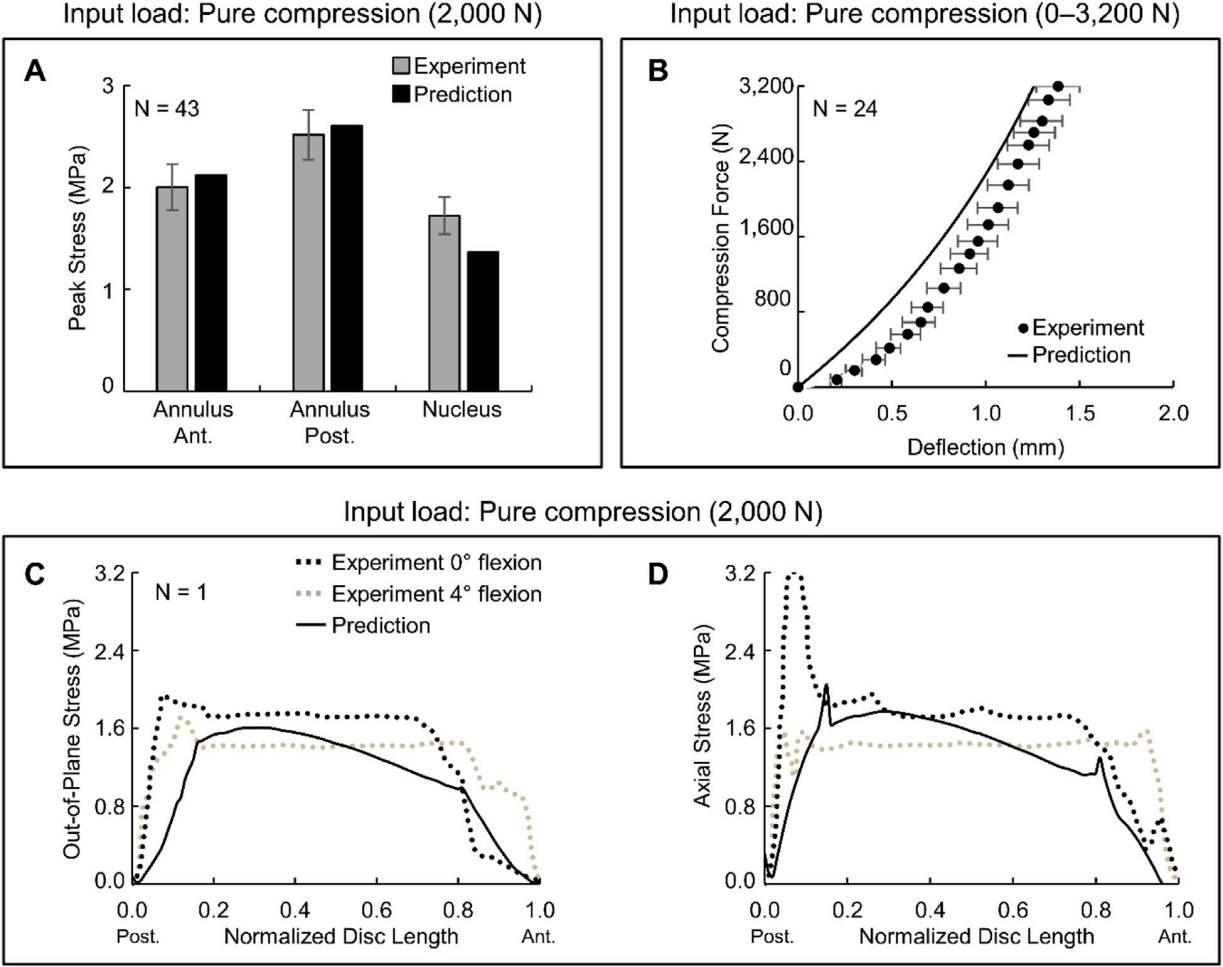
Validation studies conducted using the finite-element (FE) model of the L4-L5 lumbar segment. We assessed the ability of a participant-specific FE model (Participant P1) to predict the biomechanical responses to pure compression. **(A)** Peak stress in different regions of the L4-L5 intervertebral disc in response to a 2,000-N pure compression load. The black bar represents the FE predictions, the gray bar denotes the average of the measured peak stress (N = 43), and the vertical line represents two standard errors of the mean (SEM), for the experimental study described in (Adams et al., 1996). **(B)** Deflection at the center of the L4-L5 intervertebral disc nucleus in response to pure compression loads of different magnitude. The solid black line represents the FE predictions, the solid black circle denotes the average of the measured deflection (N = 24), and the gray horizontal line represents two SEM, for the experimental study described in (Markolf and Morris, 1974). **(C)** Out-of-plane and **(D)** axial components of stress along the sagittal midline of the L4-L5 intervertebral disc in response to a 2,000-N pure compression load. To compare the model predictions with the experimental values, we normalized the intervertebral disc length along its sagittal midline by its total length along the same midline. The solid black line represents the FE predictions. The black and gray dotted lines depict the measurements (N = 1) from the L4-L5 lumbar vertebrae with no flexion and with a 4° flexion, respectively, for the experimental study described in (Adams et al., 1994). Ant.: anterior. Post: posterior.

We validated the participant-specific FE model of the L4-L5 lumbar segment by comparing the biomechanical responses of the intervertebral disc to pure compression with experimental data from the literature (Markolf and Morris, 1974; Adams et al., 1994; Adams et al., 1996) (Figure 2). In response to pure compression, the FE model predicted higher stress in the posterior region of the annulus compared to the anterior region and the nucleus, closely mimicking the experimental trends, with differences smaller than 23% compared with the experimental values (Figure 2A). In addition, the model-predicted deflection at the center of the nucleus increased almost linearly under compression loads of up to ∼2,400 N and thereafter increased slightly nonlinearly. This differs from the experimentally measured deflection, which predominantly showed a nonlinear increase (Figure 2B). However, the root mean square error between the model-predicted values and the experimental data was a marginal 0.2 mm. Finally, we validated the predicted out-of-plane (Figure 2C) and axial (Figure 2D) stress profiles along the sagittal midline of the L4-L5 intervertebral disc in response to a 2,000-N pure compression load. The experimental profiles showed three trends: 1) higher stresses in the posterior region of the annulus (up to ∼0.2 of the normalized length), 2) relatively constant stresses along the nucleus (between 0.2 and 0.8 of the normalized length), and 3) lower stresses in the anterior region of the annulus (after 0.8 of the normalized length). In addition, the stresses decreased with an increase in the magnitude of the lumbar flexion angle (0° flexion, black dotted lines vs 4° flexion, gray dotted lines; Figures 2C,D). Except for the stress distribution along the nucleus, where our FE model predicted a decrease in stress from the posterior to the anterior region, the model reasonably predicted the overall stress distribution in the intervertebral disc.

### 3.2 Mesh convergence study

We performed mesh-convergence tests on the FE model of the L4-L5 lumbar segment to determine the adequate number of mesh elements. To this end, considering the model for Participant P1, we systematically increased the number of mesh elements in the L4-L5 intervertebral disc and evaluated the associated changes in the disc annulus stress at 0 km when the participant walked with the ExoBoot while carrying a load. The peak stress predicted at the disc annulus by the selected mesh (M4 in Table 3) increased marginally (<8%) when we increased the number of mesh elements from 9,807 to 30,744, indicating the convergence of the results.

**TABLE 3:** Summary of mesh-convergence tests performed on the finite-element model of the L4-L5 intervertebral disc.

Mesh	Number of mesh elements	Average element size (mm)	Peak stress at disc annulus (MPa)
M1	30,744	1.3	3.4
M2	21,512	1.4	3.6
M3	15,497	1.6	3.6
M4^a^	9,807	1.8	3.7

^a^ Selected.

### 3.3 Biomechanical responses of the L4-L5 lumbar segment

As described in our previous work (Nagaraja et al., 2025), when comparing changes in biomechanical responses due to walking distance, we only considered the two trials where participants carried a 22.7 kg load because one of the intended benefits of exoskeleton devices is to allow users to carry heavy loads for longer distances. We found that the device-distance interaction effect was statistically significant (*p* < 0.001) for the peak trunk flexion angle and the peak L4-L5 joint reaction force (Table 4), providing evidence that wearing the device impacts these biomechanical responses differently depending on the walking distance. For example, at baseline, participants had similar peak L4-L5 joint reaction forces when walking with the ExoBoot compared to walking without the device (ES = 0.2). In contrast, at the 5-km mark and relative to unassisted walking, participants had a lower peak L4-L5 joint reaction force when walking with the exoskeleton device (ES = −1.0). The device-distance interaction effect was not statistically significant for the L4-L5 intervertebral disc annulus stress (Table 4).

**TABLE 4:** Effect size and statistical significance of the biomechanical responses of the L4-L5 lumbar segment after walking with or without the ExoBoot for 5 km while carrying a 22.7 kg (50-lb) load.

Biomechanical response	*p*-value			Pairwise comparison							
				With vs without the ExoBoot				0 km vs 5 km			
				0 km		5 km		Without the ExoBoot		With the ExoBoot	
	Device-distance	Device	Distance	*p*-value	Effect size	*p-*value	Effect size	*p*-value	Effect size	*p*-value	Effect size
Trunk flexion angle (degree)	**<0.001**	-	-	0.438	−0.5	0.250	−1.6	0.250	1.3	0.250	0.8
L4-L5 joint reaction force (BW)	**<0.001**	-	-	0.812	0.2	0.250	−1.0	0.250	1.2	0.375	0.6
L4-L5 intervertebral disc annulus stress (MPa)	0.118	**0.022**	**0.002**	0.438	−0.2	0.250	−0.7	0.301	0.9	0.402	0.3

We evaluated the impact of walking with or without the ExoBoot (i.e., device effect), walking distance (i.e., distance effect), and their interaction on the biomechanical responses of the L4-L5 lumbar segment using linear mixed-effects models. A bold *p*-value <0.05 indicates that a categorical effect (i.e., device, distance, or device-distance interaction) significantly affected the biomechanical response. Then, we computed the effect size and performed pairwise comparisons between averages over the five participants for the two effects using the Wilcoxon non-parametric signed-rank test with Holm-Bonferroni correction. Here, a *p*-value <0.05 indicates a statistically significant difference between any two given groups. A negative effect size value indicates that participants had a lower biomechanical response when walking with the ExoBoot compared to walking without the device or that the biomechanical response decreased after walking 5 km compared to 0 km. BW: body weight.

#### 3.3.1 Effect of wearing the ExoBoot

**FIGURE 3:**
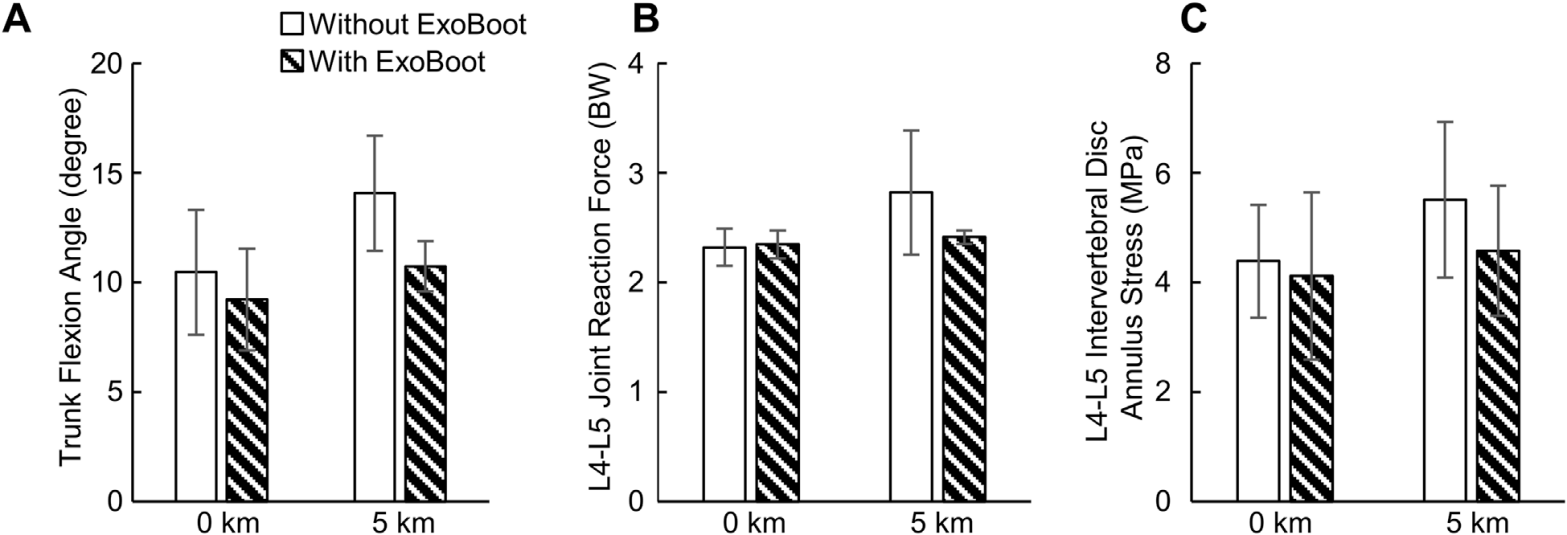
**(A)** Peak trunk flexion angle, **(B)** peak joint reaction force at the L4-L5 joint, and **(C)** peak stress at the L4-L5 intervertebral disc annulus resulting from walking with and without an active ankle exoskeleton device (the ExoBoot) while carrying a 22.7 kg (50-lb) load at 0 km and 5 km. Data are expressed as mean ± one standard deviation for N = 5 participants.

At 0 km, albeit not statistically significant compared to walking without the ExoBoot, participants had a lower peak trunk flexion angle (13%, ES = −0.5) and peak disc annulus stress (7%, ES = −0.2) when walking with the device. In contrast, the peak joint reaction force at the L4-L5 lumbar segment did not differ (<1%, ES = 0.2; Table 4; Figure 3; Supplementary Table S1). At 5 km, although not statistically significant, participants had a lower peak trunk flexion angle (27%, ES = −1.6), peak joint reaction force (15%, ES = −1.0), and peak disc annulus stress (18%, ES = −0.7) when walking with the ExoBoot compared to without the device.

#### 3.3.2 Effect of walking distance

Although not statistically significant, relative to baseline, the peak trunk flexion angle (34%, ES = 1.3), peak joint reaction force at the L4−L5 lumbar segment (22%, ES = 1.2), and peak disc annulus stress (25%, ES = 0.9) increased after participants walked for 5 km without the device (Table 4; Figure 3; Supplementary Table S1). Similarly, the peak trunk flexion angle (16%, ES = 0.8), peak joint reaction force (4%, ES = 0.6), and peak disc annulus stress (12%, ES = 0.3) increased after participants walked for 5 km with the ExoBoot.

#### 3.3.3 Stress distribution in the L4-L5 intervertebral disc

**FIGURE 4:**
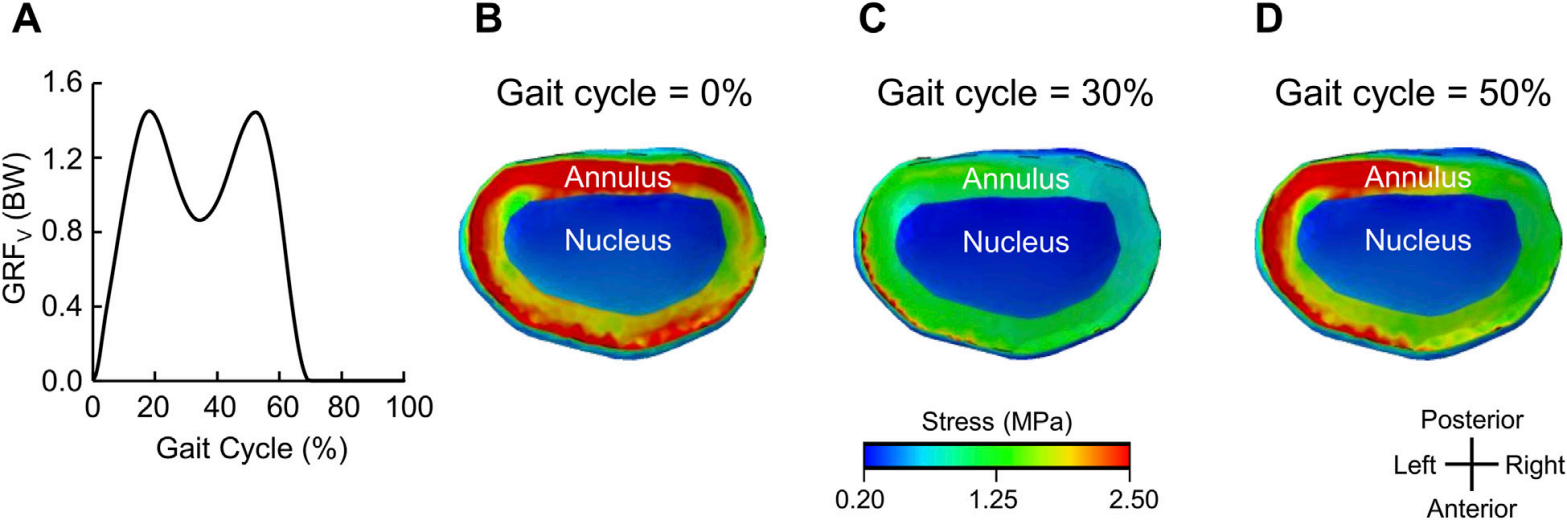
Representative stress distribution on the L4-L5 intervertebral disc for one participant (Participant P2). **(A)** Vertical ground reaction force (GRF_v_) for one representative gait cycle after walking with an active ankle exoskeleton device (the ExoBoot) while carrying a 22.7 kg (50-lb) load for 5 km. Stress distribution on the L4-L5 intervertebral disc at **(B)** 0% (heel strike and double-limb support), **(C)** 30% (midstance and single-limb support), and **(D)** 50% (terminal stance and single-limb support) of the gait cycle in panel **(A)**.

Figure 4A shows the predicted vertical GRF, and Figures 4B–D show the corresponding stress distribution in the L4-L5 intervertebral disc at different phases of the gait cycle for Participant P2 walking with the ExoBoot at 5 km while carrying a load. Except for differences in stress levels (71% with the ExoBoot and 60% without it, on average, across participants), we observed similar stress distributions for the other participants and conditions. At the beginning of the stride cycle (0%–20%), when the leading foot touched the ground, we observed uniformly distributed high stress levels along the annulus of the intervertebral disc due to the force transmitted from the ground to the body joints (Figure 4B). Next, during midstance (∼30%), we observed a 50% decrease in stress along the annulus (from 2.5 to 1.3 MPa), as the gait cycle transitioned to a single-limb support phase (Figure 4C). Finally, during the terminal stance (∼50%), we observed an asymmetrical stress distribution in the annulus, as the heel lifted off the ground and the contralateral foot began contact with the ground, suggesting an asymmetric load on the lumbar vertebrae (Figure 4D).

## 4 Discussion

The objective of this pilot study was to characterize the biomechanical responses of the L4-L5 lumbar segment in young, healthy men after walking with an active ankle exoskeleton for 5 km. To this end, we collected experimental data for five men during a 5-km walking trial while carrying a 22.7 kg (50-lb) load, both with and without wearing an active ankle exoskeleton (the ExoBoot EB60). Then, for each condition and two distances (0 km and 5 km), we developed participant-specific computational models to predict the kinematics and kinetics at the L4-L5 lumbar segment and the stress distribution in the corresponding intervertebral disc annulus. We hypothesized that the peak trunk flexion angle, L4-L5 joint reaction force, and L4-L5 intervertebral disc annulus stress would increase with an increase in walking distance irrespective of ExoBoot use. In addition, based on our previous work (Nagaraja et al., 2025), we hypothesized that exoskeleton use would mitigate these increases. In support of these hypotheses, we found that the peak trunk flexion angle and the peak annulus stress increased by 16% and 12%, respectively, after walking 5 km with the ExoBoot, and by 34% and 25%, respectively, without the device (Figure 3; Supplementary Table S1). Similarly, the peak L4-L5 joint reaction force minimally increased by 4% with the ExoBoot, while it increased by 22% without the device. Nevertheless, this new insight of the beneficial effect of active ankle exoskeleton device usage on the lumbar spine during walking with load carriage needs to be confirmed in additional investigations with a larger sample size.

As expected, we observed that the peak trunk flexion angle increased with walking distance irrespective of ExoBoot use (16% with the ExoBoot and 34% without it, Figure 3; Supplementary Table S1). This response is a compensation mechanism to propel the body forward by shifting its center of mass anteriorly and to facilitate load carriage by counterbalancing the moments around the hip and stabilizing body posture (Goh et al., 1998; Attwells et al., 2006; Aminiaghdam et al., 2017). Indeed, different studies have shown that trunk flexion increased following the onset of fatigue induced by short-interval, high-intensity running (Koblbauer et al., 2014; Winter et al., 2017; Apte et al., 2021) and plyometric exercises (Weinhandl et al., 2011; Vermeulen et al., 2023). Therefore, the increase in peak trunk flexion angle observed in our study also suggests that participants experienced some degree of fatigue by the 5-km mark. Similarly, very few studies investigated only the effect of walking distance on trunk flexion (Lidstone et al., 2017; Bloch et al., 2024). For example, Lidstone et al. (2017) measured changes in trunk flexion angle as participants walked unassisted for 5.4 km while carrying a load equivalent to 55% of their body weight and found that participants increased their trunk flexion angle throughout the walking trial, which is consistent with our findings. More recently, Bloch et al. (2024) assessed changes in trunk flexion angle during a 11.0-km unassisted ruck march and observed a marginal increase in trunk flexion and a statistically significant increase in its variability with marching distance, partially agreeing with our observation that peak trunk flexion angle increased with walking distance.

We found that the peak stress on the L4-L5 intervertebral disc annulus increased after walking 5 km (12% with the ExoBoot and 25% without it, Figure 3; Supplementary Table S1), possibly due to a redistribution of loads from the low-back muscles to the intervertebral disc, induced by changes in trunk flexion. In general, when a body joint is flexed and displaced away from its center, the surrounding muscles generate additional force to stabilize the joint (Grasso et al., 2000). In agreement with this observation, for example, we predicted increased activity in the erector spinae muscle at the 5-km mark (9% with the ExoBoot and 19% without it, Supplementary Figure S2), which we attribute to the force generated by this low-back muscle to counterbalance trunk flexion. Subsequently, as suggested by multiple studies (Dolan et al., 1994; McGill and Kippers, 1994; Colloca and Hinrichs, 2005), muscle forces generated during trunk flexion naturally redistribute from the active musculature to the various passive tissues in the lumbar region, including the intervertebral ligaments and discs, likely increasing the stress on the disc annulus, as observed in our study.

Although the peak stress on the L4-L5 intervertebral disc annulus increased after walking 5 km irrespective of ExoBoot use, the magnitude of the increase was smaller when participants wore the exoskeleton. We attribute this beneficial outcome to the reduced load on the intervertebral disc that resulted from participants maintaining a more upright trunk posture (i.e., reduced trunk flexion) when walking with the ExoBoot, possibly because they were less fatigued. We speculate that ExoBoot use helped mitigate the effect of physical exertion on trunk posture by altering lower-body biomechanics and reducing energy expenditure. For example, in the same cohort of participants, we previously found that walking with the ExoBoot was beneficial compared to unassisted walking because its use attenuated the increase in stride length (2% with the ExoBoot vs 3.5% without it) and hip extension moment (<1% vs 4%) (Nagaraja et al., 2025). In fact, studies by different groups have shown that a reduction in these two parameters lowers energy expenditure and decreases mechanical demand on the lower extremities when walking without load carriage (Leteneur et al., 2013; Antos et al., 2022). Taken together, in agreement with numerous studies on the impact of active ankle exoskeleton use on energy expenditure during walking (Mooney et al., 2014b; Zhang et al., 2017; Sawicki et al., 2020), our results suggest that the ExoBoot may reduce the energy requirement of walking with load carriage, thereby mitigating the effect of physical exertion on trunk posture and reducing the stresses on the lumbar intervertebral disc annulus.

Our finding that ExoBoot use attenuates the increase in peak stress on the lumbar intervertebral disc annulus compared to unassisted walking is clinically relevant and important for military personnel who often walk or run with load carriage and experience low-back pain (Gun et al., 2021). Indeed, different studies have associated the incidence of low-back pain with intervertebral disc degeneration (Rahyussalim et al., 2020; Mohd Isa et al., 2022). It is well established that mechanical stimulation of the disc annulus causes low-back pain (Kuslich et al., 1991) and accelerates cell-mediated remodeling events that contribute to disc degeneration (Stokes and Iatridis, 2004; Fearing et al., 2018). Therefore, by reducing the load and associated stress on the lumbar intervertebral disc annulus, ExoBoot use may help reduce the risk of intervertebral disc degeneration and low-back pain due to extended walking with load carriage.

As previously acknowledged (Nagaraja et al., 2025), our study has limitations. First, we cannot generalize the observed group-level effects because of the small sample size of this pilot study (N = 5). In addition, we limited the study to male participants because 82% of active-duty U.S. Service members are male (Department of Defense, 2023) and to eliminate sex differences that could have confounded the results. We acknowledge that there are sex-based differences in gait biomechanics when walking or running unassisted with load carriage (Bode et al., 2021; Rubio et al., 2023), and expect that some differences would persist with ExoBoot use. Second, we performed our experiments in a controlled laboratory environment using a level treadmill. Although this environment is not fully representative of U.S. Army marching, it allowed us to minimize confounding factors and systematically assess the impact of walking with the ExoBoot on the lumbar biomechanical responses. Third, we did not measure the state of fatigue of the participants. We assumed that walking 5 km at 1.3 m/s (∼60 min) with a 22.7 kg (50-lb) load would induce physical exertion, which may not be true for all participants. However, we designed our study based on a meta-analysis involving 25 different studies that identified fatigue as a likely outcome after running without a load for 1.7 km at 5.1 m/s (∼11 min) (Winter et al., 2017). Fourth, we did not account for individualized muscle strength and muscle fatigue in our musculoskeletal models. However, we individualized the tibial and L4-L5 vertebral geometry and material properties and adjusted the muscle strength based on the height, weight, and fat percentage of the participant, which added an important dimension of personalization to the models. Fifth, we did not explicitly model the collagen fibers of the annulus and the vertebral endplates in the FE model of the L4-L5 lumbar segment. Instead, similar to other computational studies (del Palomar et al., 2008; Momeni Shahraki et al., 2015), we implicitly modeled the collagen fibers using a hyperelastic, anisotropic material model and merged the vertebral endplates with the vertebrae. While the inclusion of these components would have increased the fidelity of the FE model and allowed for a more comprehensive prediction of the stress distribution in the L4-L5 lumbar segment, our validation studies suggested that these modeling assumptions likely had a non-consequential effect on the predicted displacements and stresses in the intervertebral disc (Figure 2). Finally, our findings are specific to the L4-L5 lumbar segment and additional investigations are required to more comprehensively characterize the impact of ExoBoot usage on other lumbar segments. However, we focused our study on the L4-L5 lumbar segment because it has been associated with a high risk of intervertebral disc degeneration and low-back pain (Rodriguez-Soto et al., 2017; van der Graaf et al., 2023; Bassani et al., 2024; Pav et al., 2024).

## 5 Conclusion

In this pilot study, we quantified the potential effects of an active ankle exoskeleton device (i.e., the ExoBoot) on the lumbar biomechanical responses of young, healthy men due to walking with load carriage for 5 km. Irrespective of ExoBoot use, albeit not statistically significant, we found that the peak trunk flexion angle, peak L4-L5 joint reaction force, and peak L4-L5 intervertebral disc annulus stress increased after walking 5 km, likely due to fatigue. Of note, we observed that walking with the ExoBoot reduced the magnitude of the increases in each of these biomechanical responses, which we attributed to a lower energy expenditure and the ability to maintain a more upright trunk posture compared to unassisted walking. Therefore, the results of our pilot study suggest that ExoBoot use likely attenuates the effect of fatigue on the lumbar spine induced by walking with load carriage, thereby offering potential benefits during extended use.

## Data Availability

The original contributions presented in the study are included in the article/Supplementary Material, further inquiries can be directed to the corresponding author.
