# Supplementary material for: Effect of walking with an active ankle exoskeleton on the biomechanical responses of the lumbar spine
Source: Front Bioeng Biotechnol. 2025 Sep 25;13:1654585. doi: 10.3389/fbioe.2025.1654585 (PMC12507844; doi:10.3389/fbioe.2025.1654585)
Supplement: Supplementary file 1 [file DataSheet1.docx]

***Supplementary Material***

**Effect of walking with an active ankle exoskeleton on the biomechanical responses of the lumbar spine**

Jose E. Rubio^1,2^, Junfei Tong^1,2^, Aravind Sundaramurthy^1,2^, Anup Pant^1,2^, Sridevi Nagaraja^1,2^, Meredith K. Owen^3^, Michael A. Samaan^4^, Brian Noehren^3^, and Jaques Reifman^1*^

^1^Department of Defense Biotechnology High Performance Computing Software Applications Institute, Defense Health Agency Research & Development, Medical Research and Development Command, Fort Detrick, MD, United States

^2^The Henry M. Jackson Foundation for the Advancement of Military Medicine, Inc., Bethesda, MD, United States

^3^Department of Physical Therapy, University of Kentucky, Lexington, KY, United States

^4^Department of Kinesiology and Health Promotion, University of Kentucky, Lexington, KY, United States

*Correspondence:

Jaques Reifman, Ph.D.

Department of Defense Biotechnology High Performance Computing Software Applications Institute

Defense Health Agency Research & Development

Medical Research and Development Command

ATTN: FCMR-TT, 504 Scott Street

Fort Detrick, MD 21702-5012

E-mail: jaques.reifman.civ@health.mil

Phone: + 1 301 639 3637

Fax: +1 301 619 1983

**Supplementary Table S1**. Peak trunk flexion angle, peak joint reaction force at the L4-L5 joint, and peak stress at the L4-L5 intervertebral disc annulus resulting from walking with and without the ExoBoot while carrying a 22.7-kg (50-lb) load

| **Biomechanical response** | **0 km** | | **5 km** | |
| --- | --- | --- | --- | --- |
|  | **Without the ExoBoot** | **With the ExoBoot** | **Without the ExoBoot** | **With the ExoBoot** |
| Trunk flexion angle (degree) | 10.5 (2.9) | 9.2 (2.3) | 14.1 (2.6) | 10.7 (1.1) |
| L4-L5 joint reaction force (BW) | 2.3 (0.2) | 2.3 (0.1) | 2.8 (0.6) | 2.4 (0.1) |
| L4-L5 intervertebral disc annulus stress (MPa) | 4.4 (1.0) | 4.1(1.5) | 5.5 (1.4) | 4.6 (1.2) |

The data were averaged across the five participants and are presented as mean (±1 standard deviation). BW: body weight.


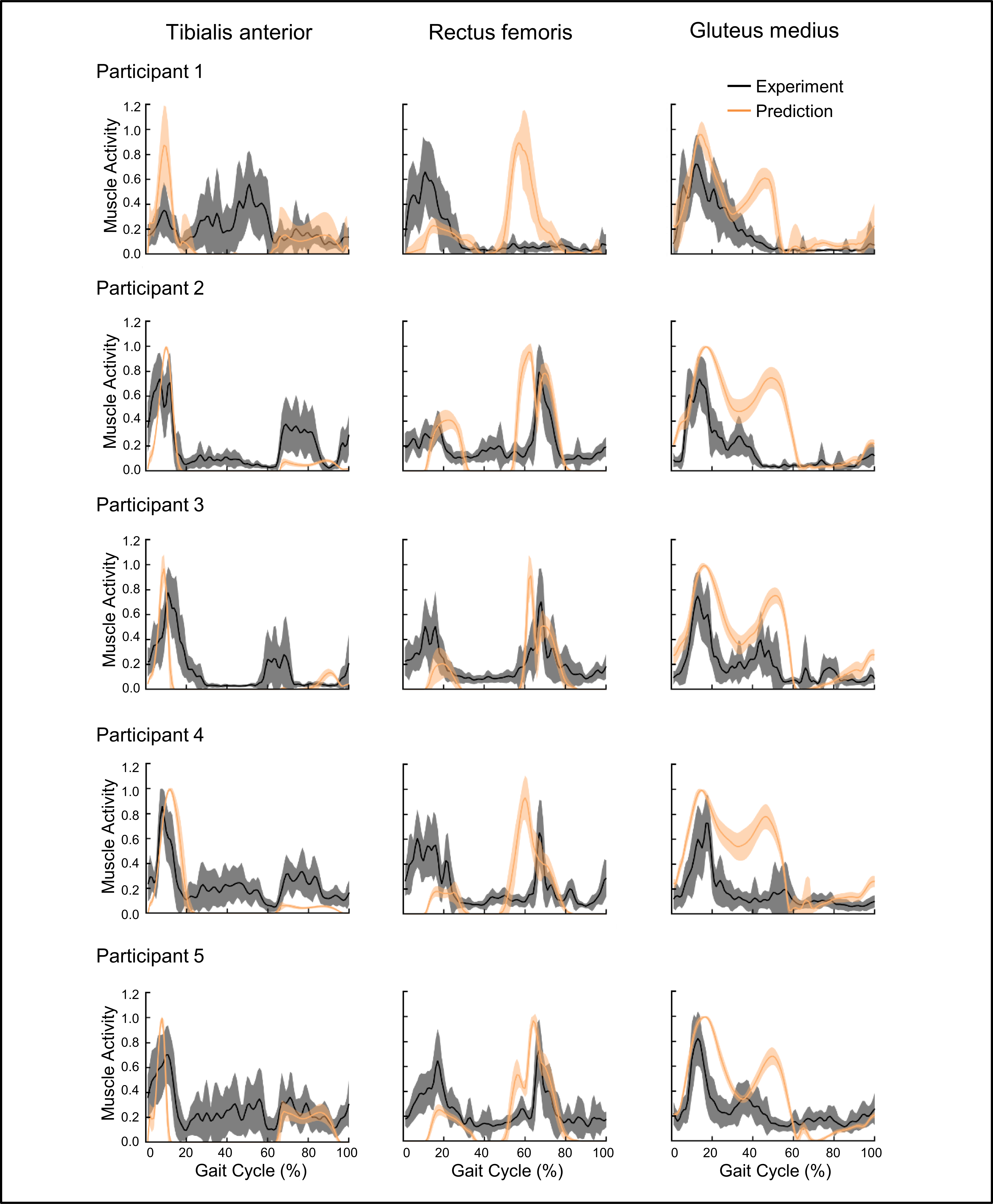


**Supplementary Figure S1**. Comparison of muscle activities predicted by the musculoskeletal model (orange solid lines) and the electromyography (EMG) data measured in the laboratory experiment (black solid lines) for the tibialis anterior, rectus femoris, and gluteus medius muscles as a function of the percent of the gait cycle for each of the five participants. The shaded areas represent the stride-averaged EMG envelopes (mean ± one standard deviation) for each participant. We separately normalized the magnitudes of the muscle activity and the EMG recordings to the maximum of each stride, for each participant.


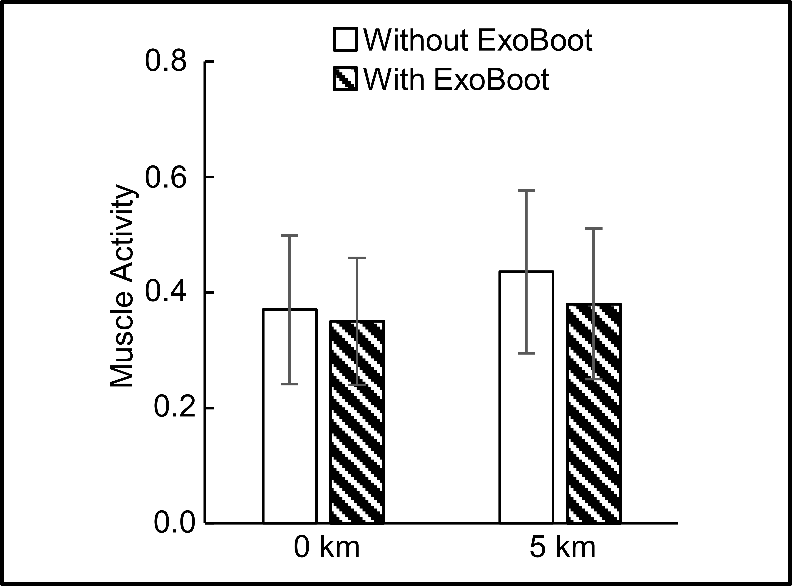


**Supplementary Figure S2**. Erector spinae muscle activity predicted by the musculoskeletal model for the conditions of walking with and without an active ankle exoskeleton device (the ExoBoot) while carrying a 22.7-kg (50-lb) load at 0 km and 5 km. Data are expressed as mean ± one standard deviation for N = 5 participants.
